# Universal Health Coverage and Primary Healthcare: Lessons From Japan: Comment on "Achieving Universal Health Coverage by Focusing on Primary Care in Japan: Lessons for Low- and Middle-Income Countries"

**DOI:** 10.15171/ijhpm.2016.120

**Published:** 2016-08-28

**Authors:** Gerald Bloom

**Affiliations:** Institute of Development Studies, University of Sussex, Brighton, UK.

**Keywords:** Universal Health Coverage (UHC), Primary Healthcare, Politics of Health

## Abstract

A recent editorial by Naoki Ikegami has proposed three key lessons from Japan’s experience of achieving virtually universal coverage with primary healthcare services: the need to integrate the existing providers of primary healthcare services into the organised health system; the need to limit government commitments to finance hospital services and the need to empower providers of primary healthcare to influence decisions that influence their livelihoods. Although the context of low- and middle-income countries (LMICs) differs in many ways from Japan in the late 19th and early 20th centuries, the lesson that short-term initiatives to achieve universal coverage need to be complemented by an understanding of the factors influencing long-term change management remains highly relevant.

A recent editorial by Naoki Ikegami draws on Japan’s historical experience to provide advice to countries wishing to move towards universal health coverage.^1^ Ikegami argues that the Japanese Government made three strategic decisions during the early stages of modernising their country’s health system, which facilitated the development of primary healthcare services: it provided opportunities for established practitioners to integrate in the organised healthcare system during a gradual transition to Western medicine; it limited the supply of hospitals and gave them little public funding and it involved the Japan Medical Association, which was dominated by clinic-based physicians, in establishing a fee schedule that favoured primary healthcare. Ikegami suggests that these measures gave providers of primary healthcare services influence over the pattern of health sector development. He acknowledges that the current situation in many low- and middle-income countries (LMICs) differs substantially from late 19th and early 20th century Japan. Nonetheless, he argues, three general lessons emerge from this experience.

## 1. Integrate Existing Providers of Primary Healthcare Services Into the Organised Health System

This is especially important in countries with pluralistic health systems, where people seek advice and obtain drugs from a wide variety of providers in terms of their training, professional standing and relationship to the regulatory system.^2,3^ In Bangladesh and India, for example, the poor make heavy use of providers of medical advice and drugs, working largely outside the regulatory framework.^4-6^ Until recently the governments of these countries ignored these providers. They allocated all public funding for primary health services to government clinics, failed to collect routine information on the informal providers and resisted any measures that could be construed as giving them “legitimacy.” The organised medical profession lobbied for this approach on the grounds of preventing a “lowering of standards.” Despite this neglect, the informal providers have continued to be the first port of call for routine health problems and recent studies have found very little difference between informal providers and private medical practitioners in their treatment of routine health problems.^7^ A recent report suggests that falls in mortality from post-natal sepsis and childhood pneumonia in Bangladesh can be largely attributed to the almost universal access to inexpensive antibiotics from informal providers.^8^ One sign of a changing approach by governments to this issue is the recent announcement by the Indian State of West Bengal of a programme of training for its rural medical practitioners. This could be a first step towards the involvement of these providers in government strategies for increasing access to primary healthcare services and towards improving the quality of services they provide.

The experience of China may point a way forward. During the period of collective agriculture in the 1960s and 1970, it trained many “barefoot doctors” to lead preventive campaigns and provide basic medical care to rural residents. During the transition to a market economy, these cadres evolved into “village doctors,” who continued to undertake some public health work, but made a living by selling drugs.^9^ The government subsequently required village doctors to pass regular examinations and register with the local health bureau. Since 2009 the government has required all local governments to allocate an agreed amount of money per capita to primary healthcare services.^10^ The village doctors are expected to play a role in the provision of these services and are paid for doing so. In many localities their work is supervised by local government health facilities and they are gradually being integrated into the primary healthcare system. However, they are not government employees and there is evidence of competition for resources between village doctors and employees of township health centres.

## 2. Limit Government Commitment to Finance Hospital Services

Ikegami acknowledges that it is much more difficult for governments to limit their financial commitment to hospital services than it was during the first half of the 20th century. Hospitals now have a much greater capacity to provide effective treatment for many serious diseases. The ageing of the population and the rapidly rising burden of illness from chronic non-communicable diseases has increased demand for hospital care. Also, the long history of unequal access to hospital care has generated political pressure to expand access to people who have been previously excluded. The experiences of South Africa and China illustrate the problem. In both countries, a privileged population group has long had access to high quality hospital services. In apartheid South Africa, the “Whites” had comprehensive health insurance that entitled them to hospital care and the hospitals gradually increased their capacity to provide modern diagnosis and treatment. In China, the employees of urban enterprises had a similar kind of coverage. During the period of rapid economic growth in the transition to a market economy, these hospitals invested substantially to provide the most up-to-date medical care. In both cases, this created a “gold standard” of sophisticated, hospital-based care to which the rest of the population aspired. Both countries have experienced a lot of pressure to extend the right to a similar standard of hospital care much more widely. Other hospitals have lobbied for improvements in their facilities, equipment and staffing and other population groups have sought access to what they perceive to be “quality care,” paying high hospital bills in many cases. The costs to individual families are often financially crippling. This has generated political pressure to protect people against “catastrophic health expenditure.”^11^ The ability of hospitals to attract the most highly trained and experienced health workers has contributed to a vicious circle in which patients bypass what they perceive to be inadequate primary level facilities and put additional pressure on hospital services. The resultant overcrowding leads to pressure to expand hospital services and neglect primary healthcare. In both countries, the governments face enormous challenges in convincing the population to seek less expensive and more appropriate primary-level services.

Governments need to find ways to protect people against catastrophic health expenditure, whilst limiting the risk of escalating hospital costs and ensuring that the population has realistic expectations of the kinds of services to which the government can ensure access. Many countries are contemplating the introduction of hospital insurance schemes in response to the financial hardship faced by families of hospitalised patients and the pressure by hospitals for additional funding. The lessons from Japan, South Africa, and China underline the need to avoid the kind of vicious circle described above. One measure that governments can take is to ensure that people have access to an effective and well-respected primary healthcare service, before guaranteeing to provide highly subsidised hospital care. They can also invest in improving the management of hospitals to ensure that they provide effective and also cost-effective services. They also need to build the capacity of government and/or insurance schemes to monitor and prevent unnecessarily rapid rises in hospital costs.

## 3. Empower Providers of Primary Healthcare Services to Ensure That Their Livelihoods Reflect Their Importance to Health and Health Services

The experiences of many countries suggest that the perspectives of hospital managers and hospital-based medical specialists tend to have undue influence on the allocation of public resources in the absence of specific measures to give voice to primary healthcare stakeholders.

In Japan, the involvement of an association of providers of clinic services in the setting of fees ensured that their interests were taken into account. Elsewhere, and in the 21st century, representatives of primary healthcare providers may be quite different. They might include primary care doctors, nurse practitioners, pharmacists, “informal providers” and community health workers. They might also include non-government organisations, faith-based organisations, and private providers of clinic services. A coalition for primary healthcare could also include representatives of users of services, such as organisations based on place of residence, ethnicity or way of making a living. In Brazil, for example, locality-based community groups have been actively involved in local health planning.^12^ The challenge for governments is to create mechanisms to enable these stakeholders to participate in decision-making and influence public opinion.

## Conclusion

The situation in many LMICs is more complex than in Japan during the first half of the 20th century. Medical knowledge and healthcare technology has advanced a lot and the expectations of people for effective care has risen. Developments in the media and information technology mean that people can compare their entitlements to those in other countries. Lobbying by health workers, providers of services and suppliers of pharmaceuticals has become sophisticated and intense. Despite these many changes, Ikegami’s emphasis on stakeholder interests and the long-term and path-dependent nature of change remains relevant. The lesson from Japan is that short-term initiatives to achieve universal coverage need to be complemented by longer-term management of health system change, which could involve building a coalition for primary healthcare; managing expectations of entitlements to health services and controlling unnecessarily large increases in public spending on hospital services. This is an important message to health sector leaders and the research community which provides evidence to support the management of change.

## Ethical issues

Not applicable.

## Competing interests

Author declares that he has no competing interests.

## Author’s contribution

GB is the single author of the paper.
